# Differences in Motor Competence of Chilean Schoolchildren According to Biological and Sociocultural Correlates

**DOI:** 10.3390/children9101482

**Published:** 2022-09-28

**Authors:** Juan Quintriqueo-Torres, Diego Menares-Quiroz, Nicolas Aguilar-Farias, Sonia Salvo-Garrido, Jaime Carcamo-Oyarzun

**Affiliations:** 1 Department of Physical Education, Sports and Recreation, Universidad de La Frontera, Temuco 4780000, Chile; 2 UFRO Activate Research Group, Universidad de La Frontera, Temuco 4780000, Chile; 3 Department of Mathematics and Statistics, Universidad de La Frontera, Temuco 4780000, Chile; 4 CIAM Physical Literacy Research Centre, Faculty of Education, Social Science & Humanities, Universidad de La Frontera, Temuco 4780000, Chile

**Keywords:** motor development, fundamental movement skills, foundational motor skills, childhood

## Abstract

(1) Background: In this study, we aimed to determine differences in the levels of motor competence according to biological factors (sex, age and weight status) and sociocultural factors (socioeconomic level and belonging to an indigenous people or not) in students of the La Araucanía Region, Chile. (2) Methods: A total of 552 students in 5th and 6th grade were evaluated (49.6% girls; age M = 11.3; SD = 0.8). To assess motor competence (domains of object control and self-movement), the MOBAK 5-6 test was applied. (3) Results: In the object control dimension, significant differences were found according to sex, with the boys performing higher than the girls. According to age, schoolchildren aged 11.0 to 11.9 performed higher than those aged 10.0 to 10.9, and according to socioeconomic status, schoolchildren from schools with a higher socioeconomic status showed a higher motor performance. No significant interaction effects were found between groups. With regard to the self-movement domain, statistically significant differences were only found according to weight status, where students of normal weight presented the highest performance. No significant effects were found between any of the groups. (4) Conclusions: This study shows the importance of considering the biological and sociocultural characteristics in the development of motor competence when interpreting data or planning interventions in different settings.

## 1. Introduction

The World Health Organization [1] considers the promotion of physical activity in children and adolescents as one of its priorities for better overall health by lowering overweight and obesity in early ages, and thus reducing the risk of this pathology in adolescence and adulthood [2,3]. Recommendations have established that children and adolescents participate in 60 min of moderate to vigorous physical activity average per day [4]. However, 80% of adolescents worldwide do not comply with this recommendation [5]. The situation is no different in Chile. According to the 2018 Report Card on Physical Activity of Children and Adolescents, only one in five children are physically active [6]. In addition, the National Survey of Physical Activity and Sport from the Chilean Ministry of Sports reports that only 16.5% of children and adolescents meet the daily minimum physical activity requirements [7].

To address this issue, it is necessary to examine the factors associated with regular physical activity. One is motor competence (MC), which along with physical fitness and the perception of competence, forms a mechanism that influences the trajectories of physical activity in childhood [8]. MC is considered a functional performance, i.e., it successfully deals with the resolution of motor tasks [9]. In this context, MC is an integral concept that considers a behavior when solving a motor task [10]. MC is a latent construct; it cannot be directly observed and is reflected through the successful completion of specific motor tasks [10]. These observable motor tasks correspond to fundamental motor skills [10], which are considered the basic requirements or “building blocks” for the accomplishment of more difficult and complex movements throughout life [11]. Considering the functionality of the fundamental motor skills, these are generally categorized as stability skills (e.g., balance), locomotion (e.g., hopping or running) and manipulation (e.g., throwing and catching) [12]. These skills develop in childhood and provide the basis for learning specific motor skills [13,14], which is why their acquisition needs to be fostered from an early age, so they are consolidated and strengthened over the years [11]. Thus, it is considered one of the main goals of physical education as a school subject [15,16] and one of the fundamental pillars for the development of physical literacy [17].

The importance of promoting the development of MC in early ages is based on evidence of the benefits associated with the acquisition and maintenance of recommended levels of physical activity [18]. Thus, adequate MC in childhood contributes to children’s physical, mental and social development, as well as to their health and well-being [19,20,21]. There is evidence of the positive association between MC and a series of outcomes in health, well-being and development, such as a healthy weight [22,23], higher levels of self-esteem [24], perceived MC [25,26], cardiorespiratory and muscular fitness [27,28,29] and reduced sedentary behavior [30], as well as improvements in cognitive development, executive functions, school preparation and academic performance [31,32]. All these benefits underscore the relevance of MC in comprehensive child development, which is why its promotion in all stages of childhood is essential [33].

MC does not develop spontaneously, but rather needs to be learned. Therefore, its development is influenced by a series of correlates, both biological and sociocultural [34], with sex, age and weight status being the most significant biological factors, while socioeconomic level is the most addressed social factor [35].

With respect to the biological factors, analyses of motor performances according to sex have shown differences in activities related to object control, where boys show higher levels of MC than girls [36,37]. However, in tasks related to self-movement, there is no definition of the role of sex, since in some studies, no associations have been made [35], and in others, girls perform better than boys [38]. With reference to age, although its increase is positively associated with MC [38], it should be noted that it is not only related to a maturational aspect, but also to the experience and interaction that children have with their surroundings [13]. In terms of the weight status, its role presents robust evidence of a negative association with MC, this being nascent in preschoolers [39,40,41] and consolidated in elementary school students [22,23], where overweight children have greater difficulties with motor tasks involving self-movement than the children of normal weight.

In relation to sociocultural factors, the socioeconomic level seems to be relevant in the development of MC, where children from vulnerable settings present lower MC and participation in physical activity [42]; however, systematic review evidence has reported inconsistent associations [35]. Object control tasks appear to be a mediator in the link between vulnerability and physical activity, as the most vulnerable children likely have less access to equipment necessary for various object control activities [42]. In addition, both the neighborhood and the school are environments for MC development; therefore, non-vulnerable neighborhoods and schools that make outdoor games accessible enable a wider range of activities and experiences that bolster motor performance [43]. This becomes relevant especially in Chile, where the schools are categorized based on their financial and administrative dependence as follows: (1) municipal schools—public schools administered by the communal governments, state property and financing; (2) subsidized schools—schools privately owned but with support from the government; and (3) private schools—privately owned schools, with private administration and financing. This categorization has wide gaps with a pronounced socioeconomic differentiation, which means that the school, instead of being a space where opportunities are equitably distributed to attenuate this differentiation, reproduces the inequalities existing in Chilean society [44]. In addition to this characteristic of the Chilean education system, other sociocultural factors inherent to the Chilean context can affect the development of MC, such as belonging to an indigenous people, a scarcely studied sociocultural factor, but with evidence in its relation to motor performance. Among the scant evidence, a study conducted with indigenous and non-indigenous students in Brazil describes children of indigenous communities as presenting higher levels of MC than their peers in an urban area [45]. In the Chilean context, this factor acquires even greater relevance, since in its territory, there are different indigenous peoples with their own culture. Against this backdrop, according to data provided by the latest national census, the Mapuche People are the largest indigenous group in Chile, with 9.9% of the total population, a proportion that reaches 34.3% in the Region of La Araucanía [46]. Despite there being some studies on MC in Chilean students [47,48,49], these have not differentiated the performance of indigenous and non-indigenous people in the analysis, so there is a need to address this topic, particularly in regions where a considerable part of the population identifies as belonging to an indigenous people.

Taking all of the above into account, not only biological factors are related to MC development, but also factors linked to the setting such as socioeconomic level and belonging to an indigenous people. Therefore, in this study, we sought to determine the differences in the levels of MC according to biological factors (sex, age and weight status) and sociocultural factors (socioeconomic level and belonging to an indigenous people or not) in students of the Region of La Araucanía, Chile.

## 2. Materials and Methods

### 2.1. Sample

A convenience sample of 552 elementary students was evaluated (49.6% girls, 50.4% boys; age M = 11.3; SD = 0.8) in 5th and 6th grades, belonging to 8 schools in 4 communes of the Region of La Araucanía, Chile. This region is located in the south of Chile, with a culturally diverse population, composed of indigenous people, mestizo and descendants of European immigrants. It is the fifth most populated region in the country [46].

The schoolchildren who participated in the study were from two municipal schools, five subsidized schools and one private school. All the participants had an informed consent agreement signed by their parents or guardians, and the children who participated signed an informed consent agreement declaring their voluntary participation in the study. The protocol used by this study was approved by the Scientific Ethics Committee of the Universidad de La Frontera (Act of Approval No. 125_17).

### 2.2. Instruments

#### 2.2.1. Motor Competence Assessment

To collect data on MC, the MOBAK 5-6 test was applied (from Motorische Basiskompetenzen in German), which was developed by Herrmann and Seeling [50] and recently validated in Spanish [47,51]. This test organizes motor competencies into two domains, one linked to object control and composed of motor tasks of throwing, catching, bouncing a ball with the hand and dribbling a ball with the foot, and another domain related to self-movement made up of the motor tasks of balancing, rolling, jumping and running (see details in Table 1). For each motor task, the students have 2 attempts (with no trials), with the exception of the tasks of throwing and catching, where six attempts must be made. The tasks are scored on a dichotomous scale (0 = failed; 1 = passed), where the number of successful attempts is recorded (never passed = 0 point; passed once = 1 point; passed twice = 2 points). In the tasks of throwing and catching, where six attempts are made, the number of hits is scored as follows: 0–2 hits = 0 point; 3–4 hits = 1 point; 5–6 hits = 2 points. Each motor task can be assessed with a minimum of zero points and a maximum of two points, so it is possible to achieve a maximum of eight points for each competence domain. The details of the procedures for the execution and assessment of the test appear in the MOBAK 5-6 Test Manual [52].

#### 2.2.2. Weight Status

Body mass index (BMI) was assessed according to the formula kg/m^2^. To determine weight status, parameters for each age and sex were considered according to the categorization by the World Health Organization [53] for children over 5 years (normal weight: BMI in percentile <85; overweight: BMI in percentile ≥85 to <97; obesity: BMI in percentile ≥97). Body weight was evaluated using a Tanita^®^ UM 2204 electronic calibrated scale (accuracy to 0.2 kg, maximum capacity 136 kg), measuring barefoot, in track pants and a t-shirt. Height was measured using a Seca^®^ 217 stadiometer (accuracy to 1 mm).

#### 2.2.3. Socioeconomic Level

The 2017 School Vulnerability Index (SVI) was used, determined by the Chilean government’s National School and Scholarship Assistance Council (JUNAEB in Spanish). The SVI is a parameter that reflects the degree of vulnerability of the students who attend a certain school, calculated considering socioeconomic factors, indicators of student performance and attendance [54]. The SVI of each school is expressed as a percentage, where a higher percentage implies a higher rate of vulnerability. Although there are no specific scales to classify the SVI, for this study, terciles were considered, where the schools with an SVI less than 33% were categorized as low, SVI between 34% and 66% as medium and SVI over 67% as high.

#### 2.2.4. Belonging to an Indigenous People

Considering that the indigenous people of the greatest prevalence in the Region of La Araucanía are the Mapuche People [46], the participants in this study were classified as belonging to this indigenous people. To classify the belonging or not to an indigenous people, the methodology of analyzing the surnames of each student was used, as is common in studies involving the indigenous population in Chile [55,56,57], where children with one or two Mapuche People surnames were classified as belonging to an indigenous people.

### 2.3. Procedure

Prior to beginning the study, informed consent was sought from the parents and the students who would participate. The assessments were applied during physical education classes. The students were asked to complete a questionnaire to compile the sociodemographic data. Then, a team of eight evaluators trained in the MOBAK battery applied the test. Each evaluator was responsible for one group of between three and five students and ran each of the assessment stations, where the motor task to perform was explained and demonstrated. Each student made two attempts (with the exception of throwing and catching, where they made six), with no trials. The approximate duration of the test application was 45 min.

### 2.4. Data Analysis

The SPSS v. 25 data statistics program was used for the analysis (IBM Corp, Armonk, NY, USA). Descriptive statistics analyses were performed using central tendency and dispersion. The normality of the data was determined by the standardized asymmetry coefficient and kurtosis [58]. To determine possible differences in levels of MC according to sex, age group, weight status, socioeconomic level and indigenous or non-indigenous status, as well as to determine the interaction effects of these variables, a Univariate General Linear Model was fitted, which provides a regression analysis and an analysis of variance for a dependent variable using one or more factors [59]. For variables with 3 groups (weight status and socioeconomic status) the Bonferroni statistic was used as a post hoc test. The effect size was determined by *η*^2^*_p_*, considering as trivial, small, moderate, or large the values smaller than 0.01, between 0.01 and 0.06, between 0.06 and 0.14, and higher than 0.14, respectively [60]. The α level of statistical significance was set at *p* ≤ 0.05.

## 3. Results

Table 2 shows the analyses of variance for the object control domain according to each group. Significant differences were found according to sex (*F*(1, 539) = 15.595; *p* = 0.001; *η*^2^*_p_* = 0.022), where the boys outperformed the girls. Significant differences also exist according to age group (*F*(1, 539) = 3.475; *p* = 0.032; *η*^2^*_p_* = 0.014), particularly between the age groups 11.0 to 11.9 years and 10.0 to 10.9 years (*p* = 0.016). Significant differences were also found according to socioeconomic level (*F*(1, 539) = 3.475; *p* = 0.032; *η*^2^*_p_* = 0.014), specifically between students of schools with a low level of vulnerability and a high level of vulnerability (*p* = 0.032). No significant interaction effects were found between groups.

Table 3 shows the analyses of variance for the self-movement dimension. When comparing the groups, statistically significant differences were only found according to weight status, where children with a normal weight status had the highest values, followed by overweight students and then by obese students. Significant differences were found (*F*(2, 539) = 5.195; *p* = 0.006), where post hoc analyses indicated differences between normal weight and overweight schoolchildren (*p* = 0.004), between normal weight and obese schoolchildren (*p* < 0.001) and between overweight and obese schoolchildren (*p* = 0.042). Regarding the interaction effects between all groups, no significant effects were found between any of the groups.

## 4. Discussion

We endeavored to determine the differences in MC levels according to biological factors (sex, age and weight status) and sociocultural factors (socioeconomic level and belonging or not to an indigenous people) in students in the Region of La Araucanía, Chile.

With reference to the biological factors, specifically to sex, the results of this study elucidate differences between both sexes in handling objects, where the boys outperformed girls; however, in self-movement, no differences were found. This is consistent with the current evidence that boys perform higher than girls in the domain of object control [35,36,38]. For the domain of self-movement, the results of this study are along the same lines as those reviewed by Barnett et al. [35], who found no evidence that the students’ sex is associated with self-movement-related tasks. Nevertheless, it should be pointed out that other studies have indeed found a link between student’s sex and motor tasks related to self-movement [38]. Despite these disparities, sex plays an important role in MC, which may be attributable to students’ experiences of movement, considering that there are certain reinforcements of particular types of activities related to being a boy or a girl [61]. Thus, boys receive greater support and opportunities to participate in certain physical activities, especially in sports with a ball, whereas girls perform other activities such as individual sports or activities such as dance [62,63]. It is even posited that this differentiation is reinforced in physical education classes, since activities usually focus on meeting the expectations of boys, oriented to games with a ball [64]. Consequently, physical education classes are dominated by activities that are commonly requested by boys, while activities that are usually more preferred by girls, such as dance and gymnastics, are marginalized [65]. Thus, the role of teachers is essential to change these stereotypes, as they can either promote attitudinal changes or continue to reproduce these differences [66].

With regard to the age variable, although in the present study, the older students presented higher values of MC, significant differences were found only in the domain of object control. Several studies have found that increasing age is related to MC, where motor performance in certain tasks such as balancing, throwing and catching is positively related to chronological age [35,67,68]. This increase is produced by both maturational factors and movement experiences resulting from interactions with the environment [13]. In our study, the differences between age groups were not strong, which may be caused by the fact that the participants belonged to grades 5 and 6, where the three age groups are distributed in only two grades, so that students of different age groups might be in the same grade. Therefore, sixth graders presented greater motor experiences than fifth graders.

In terms of weight status, the results show there are no differences in the domain of object control, whereas in the domain of self-movement, all the groups differed from each other, with the students of normal weight presenting the highest values, followed by the overweight students, and finally the students with obesity having the lowest performance. There is strong evidence of a negative association between weight status and MC, where the children with overweight or obesity have greater difficulties with motor tasks that involve self-movement than children of normal weight [22,23,69]. Overweight and obese schoolchildren have to make motor adaptations in order to participate in physical activities, as an increased mass leads to changes in posture and decreased balance [67]. A high BMI makes it more difficult to balance or even to move around [22], which would explain the difficulties in solving motor tasks involving self-movement, whereas object control tasks can be performed without the necessity of displacement, which may mask the lower MC of overweight and obese children [70]. The negative relationship between weight status and MC increases the difficulties for children with overweight or obesity to participate in their MC development, which becomes a downward spiral by reducing the possibilities of performing physical activity [8,14]. For this reason, it is necessary to pay special attention to schoolchildren who are overweight and obese, especially those who do not practice any type of structured PA, in order to take measures to promote MC [22].

With regard to the sociocultural factors, when analyzing the findings referring to socioeconomic level, the students from schools with low vulnerability, i.e., a better socioeconomic level, had higher motor performances. These findings are in line with several studies [42,43,71] that point to the existence of inequality in the development of MC, where socially underprivileged children perform more poorly than socially advantaged children. The students who attend schools with low vulnerability belong to the families with greater economic resources, so they have access to more types of extracurricular physical activity programs [72,73]. Moreover, the type of school could directly affect the promotion of tools for physical recreation, with the private system (associated with low vulnerability) being the one that has access to larger amounts of playground space and greater offerings of physical activities. By contrast, for the students who attend public schools (associated with high levels of vulnerability), there seems to be less access to playground space, and fewer physical activities are offered [74]. This evidence is also manifest in the Chilean context, since in the 2016 Report Card on Physical Activity [75], the link between socioeconomic level and the practice of physical activity and sports was also noted, which could explain part of these differences, since children from lower socioeconomic levels have fewer opportunities to learn and put their MC into practice.

In reference to belonging or not to an indigenous people, in the domains of object control and self-movement, the study participants did not present any significant differences. This diverges from the results obtained by Duarte et al. [45], who found that the indigenous children had better motor performance than non-indigenous students. This discrepancy in the results could be due to the geographic composition of the sample, since in the study by Duarte et al. [45], the participants who belonged to indigenous people lived in rural towns, whereas in our study, all the participants lived in urban areas. In the case of children from rural indigenous communities, their level of mobility, their opportunities to explore the context and the availability of time to play are more frequent than in non-indigenous communities [76], which may explain the results reported by Duarte et al. [45]. In our case, those who belong to indigenous people and those who do not are in the same schools, are classmates and receive the same educational tools and motor experiences.

This study has some limitations. The sample of participants, as a convenience sample concentrated in four cities in the Region of La Araucanía in Chile, is not representative of the population, and we cannot extrapolate the results to the entire Chilean schoolchildren. It is recommended that future studies include not only students from an urban population, but also students from rural areas, delving more deeply into a scarcely studied topic [45]. It is also recommended that belonging or not to indigenous people be not only defined on the basis of the participants’ surnames, but also the alternative of declaring oneself as belonging to an indigenous people because one feels part of them, following national guidelines [77]. Finally, for future studies, it is recommended to consider aspects such as complementing the age variable with the maturational peak, which will allow a better determination of the role of this variable in the motor performance, and also the assessment of the participants’ physical activity, as this would offer a better understanding of its relationship with MC, and biological and sociocultural characteristics could mediate this relation.

## 5. Conclusions

This study shows the importance of considering biological and sociocultural characteristics in the development of MC, highlighting the differences that exist in the same group of elementary students. As a biological factor, sex is a correlate in MC, mainly in object control, where it is confirmed that boys score higher than girls. The increase in age is also a correlate of children’s MC. Furthermore, weight status is another important determinant in the performance of motor tasks that involve locomotion, presenting a negative association with MC to the detriment of students who are overweight and obese. Sociocultural factors, especially socioeconomic level, are other relevant features in the MC developmental process, with inequalities appearing according to the students’ level of vulnerability. As in other aspects of society, these inequalities might hamper children from families with limited means. Belonging or not to an indigenous people revealed no differences in the motor performance of students in urban areas; hence, it would not be a determinant in their development. However, as the evidence in the international literature is not abundant, this topic should be investigated further. It is important to continue this line of research and to deepen the study of these characteristics so as to understand how their role affects the development of motor competence.

## Figures and Tables

**Table 1:** Description of the items in the MOBAK 5-6 test [26].

Item		Description
Object Control	Throwing	The child throws six juggling balls at a target from a distance of 3.5 m.
	Catching	The child throws a tennis ball at a wall from a scratch line at a distance of 3.0 m. The child catches the tennis ball directly from the air when it bounces back.
	Bouncing	The child bounces a basketball (size 6) back and forth through a marked corridor (8.0 × 1.1 m) with four obstacles of 0.7 m width, without losing the ball.
	Dribbling	The child dribbles a futsal ball (size 4) back and forth through a marked corridor (8.0 × 1.1 m) with four obstacles of 0.7 m width, without losing the ball.
Self- movement	Balancing	The child balances back and forth over an overturned long bench placed on a springboard, passing two obstacles taped to the bench without touching them.
	Rolling	The child performs a forward roll, starting with a jump over a set-up banana box.
	Jumping	The child skips a rope for 20 s, changing rhythm after 10 s.
	Running	The child moves forward and sideways along a square (4.0 × 4.0 m) marked on the floor. While running forward, the child jumps through three evenly spaced hoops lying on the floor.

**Table 2:** Results for the object control domain according to sex, age, weight status, socioeconomic level and belonging or not belonging to an indigenous people.

		Object Control			*F*	*p*	*η* ^2^ *_p_*	*Sig. between*
		n	M	SD				
*Biological factors*								
Sex								
a.	Girls	268	2.5	1.90	10.595	0.001 **	0.022	-
b.	Boys	272	3.8	2.05				
Age								
a.	10.0–10.9 years	168	2.8	2.08	3.475	0.032 *	0.014	a-b
b.	11.0–11.9 years	278	3.3	2.11				
c.	12.0–13.0 years	94	3.2	1.97				
Weight status								
a.	Normal weight	186	3.4	2.11	1.592	0.205	-	-
b.	Overweight	175	3.0	2.06				
c.	Obesity	179	3.1	2.07				
*Sociocultural factors*								
Socioeconomic level								
a.	Low vulnerability	53	3.8	1.98	3.478	0.032 *	0.014	a-c
b.	Medium vulnerability	33	3.8	2.10				
c.	High vulnerability	454	3.0	2.08				
Indigenous people								
a.	Belonging	98	3.3	2.21	0.032	0.859	-	-
b.	Not belonging	442	3.1	2.06				

M = mean, SD = standard deviation. * Significant differences at the level of *p* ≤ 0.05. ** Significant differences at the level of *p* ≤ 0.001.

**Table 3:** Results for the self-movement domain according to sex, age, weight status, socioeconomic level and belonging or not belonging to an indigenous people.

		Self-Movement			*F*	*p*	*η* ^2^ *_p_*	*Sig. between*
		n	M	SD				
*Biological factors*								
Sex								
a.	Girls	268	2.65	2.04	0.096	0.757	-	-
b.	Boys	272	2.48	1.93				
Age								
a.	10.0–10.9 years	168	2.31	2,02	1.560	0.211	-	-
b.	11.0–11.9 years	278	2.65	1.99				
c.	12.0–13.0 years	94	2.80	1.88				
Weight status								
a.	Normal weight	186	3.15	1.95	5.195	0.006 *	0.021	a-b; a-c; b-c
b.	Overweight	175	2.54	1.98				
c.	Obesity	179	1.98	1.87				
*Sociocultural factors*								
Socioeconomic level								
a.	Low vulnerability	53	3.26	2.10	1.546	0.214	-	-
b.	Medium vulnerability	33	2.06	1.73				
c.	High vulnerability	454	2.52	1.98				
Indigenous people								
a.	Belonging	98	2.35	2.03	0.334	0.564	-	-
b.	Not belonging	442	2.61	1.98				

M = mean, SD = standard deviation. * Significant differences at the level of *p* ≤ 0.05.

## Data Availability

The underlying research materials related to this paper are available from the corresponding author upon request.
